# Creation of a Holistic Platform for Health Boosting Using a Blockchain-Based Approach: Development Study

**DOI:** 10.2196/44135

**Published:** 2023-04-19

**Authors:** Juan Lopez-Barreiro, Luis Alvarez-Sabucedo, Jose-Luis Garcia-Soidan, Juan M Santos-Gago

**Affiliations:** 1 Faculty of Education and Sport Sciences University of Vigo Pontevedra Spain; 2 AtlanTTic University of Vigo Vigo Spain

**Keywords:** blockchain, exercise, gamification, habits, healthy lifestyle, physical fitness

## Abstract

**Background:**

Low adherence to healthy habits, which is associated with a higher risk of disease and death, among citizens of Organization for Economic Co-operation and Development countries is a serious concern. The World Health Organization (WHO) and the physical activity (PA) guidelines for Americans provide recommendations on PA and healthy diets. To promote these habits, we suggest using a blockchain-based platform, using the PA Messaging Framework to deliver messages and rewards to users. Blockchain is a decentralized secure platform for data management, which can be used for value-added controls and services such as smart contracts (SCs), oracles, and decentralized applications (dApps). Of note, there is a substantial penetration of blockchain technologies in the field of PA, but there is a need for more implementations of dApps to take advantage of features such as nonfungible tokens.

**Objective:**

This study aimed to create a comprehensive platform for promoting healthy habits, using scientific evidence and blockchain technology. The platform will use gamification to encourage healthy PA and eating habits; in addition, it will monitor the activities through noninvasive means, evaluate them using open-source software, and follow up through blockchain messages.

**Methods:**

A literature search was conducted on the use of blockchain technology in the field of PA and healthy eating. On the basis of the results of this search, it is possible to define an innovative platform for promoting and monitoring healthy habits through health-related challenges on a dApp. Contact with the user will be maintained through messages following a proposed model in the literature to improve adherence to the challenges.

**Results:**

The proposed strategy is based on a dApp that relies on blockchain technology. The challenges include PA and healthy eating habits based on the recommendations of the WHO and the Food and Agriculture Organization. The system is constituted of a blockchain network where challenge-related achievements are stored and verified using SCs. The user interacts with the system through a dApp that runs on their local device, monitors the challenge, and self-authenticates by providing their public and private keys. The SC verifies challenge fulfillment and generates messages, and the information stored in the network can be used to encourage competition among participants. The ultimate goal is to create a habit of healthy activities through rewards and peer competition.

**Conclusions:**

The use of blockchain technology has the potential to improve people’s quality of life through the development of relevant services. In this work, strategies using gamification and blockchain are proposed for monitoring healthy activities, with a focus on transparency and reward allocation. The results are promising, but compliance with the General Data Protection Regulation is still a concern. Personal data are stored on personal devices, whereas challenge data are recorded on the blockchain.

## Introduction

### Background

In modern societies, many of the deaths and diseases that occur could easily be avoided if people adopt healthy lifestyle habits [1-4]. Therefore, the governments of the Organization for Economic Co-operation and Development (OECD) countries are especially interested in promoting healthy lifestyle habits among their citizens and have been making relevant policies. The problem observed in these policies, however, is the low adherence to these habits among the general population. It seems, therefore, that the difficulty lies not in defining these habits but in generating a culture based on them.

On the one hand, recommendations for the practice of PA and its benefits for people’s health, based on scientific evidence, can be found in the physical activity (PA) guidelines of the World Health Organization (WHO) [1] and the PA guidelines for Americans (PAG) [2]. These guidelines state that, in general, for all populations, some exercise is better than none. If people who do not practice any PA just start doing so, they will obtain health benefits. It is recommended that people with sedentary habits should perform PA following the principles of load progression [5]. People who perform moderate-intensity PA can gradually begin performing vigorous PA. In addition to the practice of PA, a healthy diet is recommended, which involves reducing sugar, fat, and salt consumption and limiting the consumption of processed foods and foods containing saturated fats.

On the other hand, the guidelines recommend the consumption of fruits, vegetables, legumes, nuts, and whole grains, as well as the consumption of at least 5 servings of fruits and vegetables daily [6-12]. The guidelines also emphasize that poor dietary habits together with a lack of PA greatly increase the risk of contracting noncommunicable diseases [6,11].

This is why it is of great social value to provide tools to promote these healthy habits in the population and monitor adherence. To this end, this paper explores the use of a platform to promote these habits and monitor adherence taking advantage of blockchain (BC) for gamification techniques. To carry out this gamification, the Physical Activity Messaging Framework [13] is used to organize the delivery of the most appropriate messages and rewards to the user to encourage their participation. These messages are categorized as generic, targeted, tailored, tailored personalized, and generic personalized, and as we progress through them, they become more relevant and more personal to the user.

Generic messages are those that apply to any person in general, regardless of their particularities, and that inform about the benefits of PA practice (eg, “Performing PA is good for your health”) [14]. By contrast, targeted messages are relevant to a specific group [15], in this case, the general population of adults, and specifically highlight the benefits of PA practice in this group (eg, “Adults should perform 30 minutes of moderate PA per day to improve their cardiovascular health”). To engage the user in a more personal way, tailored messages are used. These messages use specific data about each individual user (eg, their goals) to make the message more relevant [15] (eg, “You are only 10 minutes away from reaching your weekly PA goal. Achieve it and improve your cardiovascular health!”).

A personalization layer can be added to these messages, which consists of adding data that are not related to PA, such as the name of the user, to increase the salience and proximity of the message [15]. Thus, this feature can be added to generic messages (eg, “Hi Manuel! Doing PA is good for your health”) and to tailored messages (eg, “Hi Rosa! You are only 10 minutes away from reaching your weekly PA practice goal. Achieve it and improve your cardiovascular health!”).

In addition, messages can be classified according to whether they are framed to highlight the benefit of meeting the proposed objectives (gain framed; eg, “PA practice reduces the risk of heart disease, hypertension, and type 2 diabetes”) or to point out the harms of not meeting them (loss framed; eg, “Not performing PA increases the risk of heart disease, hypertension, and type 2 diabetes”) [16]. Messages aimed at highlighting the benefits of performing PA are generally recommended to promote PA practice [14,16]. By contrast, messages emphasizing the harms of not performing PA may also be recommended in certain cases, such as back injuries, where it may be beneficial to increase the perceived risk of not performing PA to engage users [17,18].

BC is a technology that provides features such as decentralization, transparency, open source, autonomy, immutability, and anonymity [19]; it can be conceptualized as a new model for the externalization of trust in information management in a distributed environment [20]. It consists of generating a general ledger, in which, using accounting terminology, the information is stored. This information, by the nature of the system itself, becomes immutable. To this end, it relies on a *peer-to-peer* structure in which the nodes or members participating in the system collaborate with each other to guarantee the inviolability of the data and their high availability, subject neither to the failure of a server nor to the management of a third party. This latter aspect is what allows it to become the appropriate tool when it is not desirable to rely on third parties. The nodes within the network themselves validate the records and add them to a chain of blocks (hence the name of the technology), which constitutes the aforementioned ledger of records [21].

When an agent wishes to enter a new record in this ledger, the agreement of all members of the ledger’s host network is needed before the record can be validated. This is done by using a specific protocol called a *consensus algorithm*, which establishes the criteria for the acceptance of an element in the chain of records. The 2 most common consensus algorithms are *proof of work* (PoW) [22], used in the bitcoin network, whereby miners must solve a complex mathematical problem to justify the inclusion of the new block, and *proof of authority* (PoA) [22], which allows the inclusion of new records based on the relevance of the miner making the proposal. These algorithms are only a small sample of the plethora of proposals in the literature.

This technology is achieving high market penetration as a solution for information storage and verification in a wide variety of domains, ranging from cryptocurrencies to the traceability of food and pharmaceutical products [23,24]. As shown in a recent systematic review [25], a significant penetration is observed in the field of PA but with a poor leveraging of the special features that BC offers to implement value-added controls and services such as smart contracts (SCs), oracles, and decentralized applications (dApps), each of which is described in Textbox 1.

**Descriptions of smart contracts (SCs), oracles, and decentralized applications (dApps).**

- SCs: these are executable codes that run on top of the blockchain to facilitate, execute, and enforce an agreement among untrustworthy parties without the involvement of a trusted third party. SCs provide network automation and the ability to convert paper contracts into digital contracts [26].
- Oracles: SCs cannot take into consideration information that is not registered in the network itself. To alleviate this shortcoming and to improve the functionality of the network in general, oracles are introduced. Oracles are responsible for registering data captured from the real world in the network without the intervention of a human user [27].
- dApps: these are applications that run on one or more clients using information hosted in a distributed manner in a blockchain network, taking advantage of the characteristics of these networks. By making use of SCs, operations and verifications of conditions (such as those imposed in a challenge in a gamification context) are carried out without any human intervention [28].

As reported by Cai et al [28], the implementation of dApps is required to exploit another important feature existing within this environment, namely nonfungible tokens (NFTs). An NFT is an encrypted digital asset, a special type of cryptographic token that represents something unique. NFTs serve to prove that a certain user is in possession of a token that is unique, traceable, and exchangeable; they are very useful in certain gamification contexts to reward users for achieving their goals [29].

### Objectives

On the basis of the points outlined so far, this work proposes the creation of a platform to facilitate the inculcation of healthy lifestyle habits and practices through a gamification strategy. The objective was to engage users—society as a whole—in activities that can become healthy habits. These healthy habits will be both sporting and nutritional.

One of the highlights of this platform is its great potential in terms of gamification. This tool provides a very functional support for the monitoring of the data hosted on it without human intervention (eg, the validation of the challenges presented to the users).

In addition, within this platform, the possibility of defining challenges in a highly parameterized way is contemplated so that different state or private agencies can, in due course, propose their own challenges and make them available to users.

We can say, therefore, that the objective of this work is to present a holistic platform for the support of health-related challenges among the population, using scientific evidence with the support of BC technology. It is intended to provide a mechanism to encourage and set healthy PA and eating habits among the general population by using gamification techniques through challenges.

This platform will integrate the noninvasive monitoring of the proposed activities, evaluation through open-source software, and follow-up using BC through messages addressed to the end user that will allow them to adhere to this activity.

## Methods

As a first step, the state of the art in this regard was checked, through a literature search, to get an idea about the use of BC technology in the field of PA and healthy eating [25]. On the basis of the results of this search, it is possible to define an innovative platform for the promotion and monitoring of healthy lifestyle habits based on scientific evidence. The goal is to introduce healthy habits concretized in activities defined within different health-related challenges through the use of a dApp for the general population.

One of the key aspects with regard to improving users’ adherence to the training program embedded in the challenges is to maintain contact with the user. To organize this information delivery to the user, messages will be used following the model proposed in the study by Williamson et al [13].

## Results

The aforementioned review of the current literature shows a significant increase in scientific production related to BC technology in recent years, as is also indicated in 2 bibliometric reviews [24,30]. Among the large number of existing works that take advantage of this technology, the following are worth mentioning.

### BC and PA and Health Care

In the literature, it is possible to find several works that combine BC technology with PA practice and health care. Among them is the study by Alsalamah et al [31], who proposed a platform to incentivize PA practice and encourage a healthy lifestyle through gamification and rewarding of users for meeting their goals using cryptocurrencies. Another noteworthy study is the one by Frikha et al [32], who stored users’ health data in electronic health records to diagnose and treat patients more easily and cost-effectively. Other notable works are those by Jamil et al [33] and Jamil et al [34]; the authors assigned training and diet programs to each user based on their anthropometric and body composition data. Furthermore, in the study by Jamil et al [34], the authors allowed the transfer of the user profile among different sports centers.

### BC and Sport

Other trending works have made contributions that are restricted to the sporting field, ranging from data capture and management to predictions of sporting performance. This is the case with the study by Cao et al [35], who developed a model to predict performance and improve training; the study by Hong and Park [36], who captured players’ performance data to make tactical decisions in situ and in real time; and the study by Yu [37], who developed a model to improve and guide training using athletes’ physiological data. Moreover, we have the study by Ma [38] in which the author filtered data from users’ gait patterns; as well as the study by Shan and Mai [39], who proposed a system to capture and manage athletes’ fitness data in real time. Finally, there is the study by Mulyati et al [40] in which a model was developed to store data regarding belt promotions and grades in taekwondo, bringing transparency and immutability to the scores.

### BC and Active Aging

There are also very diverse contributions related to the incorporation of BC into active aging. Khezr et al [41] developed a system that provides alerts when normal behavioral patterns change. Rahman et al [42] assigned therapies based on users’ treatment needs. Rahman et al [43] developed a system to control smart home devices using gestural recognition tools. Rupasinghe et al [44] determined the risk factors for falls and developed a model to predict them. Silva et al [45] captured physiological data of patients and made them secure and interoperable through BC. Spinsante et al [46] proposed an app to promote active aging and assess the level of PA practice and quality of life. Finally, Velmovitsky et al [47] proposed a system for users to control informed consent for their participation in studies at all times.

Table 1 shows a synthesis of the state of the art in different technological aspects, such as the use of SCs and oracles, support for cryptocurrencies and NFTs, training and dietary programs based on scientific evidence, and end-user delivery support.

**Table 1:** Analysis of studies related to blockchain and physical activity and health care, sport, and active aging.

Domain and reference		SC^a^	Oracle	Cryptocurrencies	NFT^b^	Evidence based	End-user delivery support
**Physical activity and health care**							
	Alsalamah et al [31]	Yes	No	Yes	No	No	Web dApp^c^ and mobile app
	Frikha et al [32]	Yes	No	No	No	No	Web application and mobile app
	Jamil et al [33]	Yes	No	No	No	No	Web application
	Jamil et al [34]	Yes	No	No	No	No	Web application
**Sport**							
	Cao et al [35]	No	No	No	No	No	Not described
	Hong and Park [36]	No	No	No	No	No	Not described
	Ma [38]	No	No	No	No	No	Not described
	Mulyati et al [40]	No	No	No	No	No	Web application and mobile dApp
	Shan and Mai [39]	No	No	No	No	No	Not described
	Yu [37]	No	No	No	No	No	Not described
**Active aging**							
	Khezr et al [41]	Yes	No	No	No	No	Not described
	Rahman et al [42]	Yes	No	No	No	No	Not described
	Rahman et al [43]	Yes	No	No	No	No	Web application and mobile dApp
	Rupasinghe et al [44]	Yes	No	No	No	No	Not described
	Silva et al [45]	No	No	No	No	No	Web application and mobile app
	Spinsante et al [46]	No	No	No	No	No	Web application and mobile app
	Velmovitsky et al [47]	Yes	No	No	No	No	Not described

^a^SC: smart contract.

^b^NFT: nonfungible token.

^c^dApp: decentralized application.

The studies cited (Table 1) dealt with the introduction of BC in the field of PA and health care, sport, and active aging. However, most of them (14/17, 82%) show very limited development, which shows us the initial stage of development of this technology in the field concerned. Only 9 (53%) of the 17 studies make use of SCs [31-34,41-44,47]. Among those describing the access policy, most (10/17, 59%) use private and authorized networks; of the 17 studies, only 1 (6%) uses a public network, and 1 (6%) uses an authorized consortium. In addition, only the study by Alsalamah et al [31] incentivizes using cryptocurrencies as a reward. None of the cited works make use of NFTs, and none base their training or dietary plans on scientific evidence. Regarding the delivery medium, most used web applications or mobile apps, and only 3 (18%) of the 17 studies leveraged dApps [31,40,43].

On the basis of this review of the state of the art and relying on the Physical Activity Messaging Framework and BC technology, challenges will be proposed to the general population and monitored through the use of a dApp that relies on the information and SCs stored in the BC.

These challenges are composed of (1) a series of PAs and specific healthy eating habits that generate benefits for the user when performed with the proposed sequencing and periodicity and (2) the messages corresponding to each challenge.

The PAs included in these challenges are based on scientific evidence following the recommendations for the general adult population found in the PA guidelines of the WHO [1] and the PAG [2], whereas the proposed healthy eating habits are based on the recommendations of the WHO and the Food and Agriculture Organization (FAO) [7,11]. We list here in a concrete and clear way the PA practice recommendations for the general adult population that will be the basis for the subsequent creation of the different challenges that users will have to complete to obtain their rewards (it is recommended to exceed the upper limits of moderate and vigorous PA or perform a combination of both):

- Moderate PA per week: 150 to 300 minutes
- Vigorous PA per week: 75 to 50 minutes
- Strength PA per week: ≥2 sessions

Among the aforementioned recommendations, we find different PA modalities such as aerobic exercise (muscle movement in a rhythmic way and maintained over time), muscle strengthening (strength training and weight lifting), bone strengthening (produces a force in the bones that promotes their growth and strength), balance training (improves the ability to resist internal or external forces of the body that cause falls), and multicomponent training (a combination of aerobic PA, balance training, and muscle strengthening), which will bring some benefit to the user when performed [2]. Of note, Momma et al [48], in their recent systematic review and meta-analysis of cohort studies on muscle-strengthening activities, highlighted the reduction in the risk of all-cause mortality, cardiovascular disease, cancer, and diabetes in participants by 10% to 17% [48]. Regarding healthy eating habits, the WHO recommends restricting sugar consumption to <10% of total daily calories, fat consumption to <30% of total daily calories, and salt consumption to <5 g daily, as well as limiting the consumption of processed foods and foods containing saturated fats to <10% of total calorie intake and foods containing trans fats to <1% of total calorie intake. By contrast, the guidelines recommend the consumption of fruits, vegetables, legumes, nuts, and whole grains, as well as the consumption of at least 5 servings of fruits and vegetables daily [6-12]. Aune et al [6] report a 31% decrease in the risk of contracting diseases with a daily intake of 800 g of fruits and vegetables, a 19% decrease with a daily intake of 600 g of fruits, and a 25% decrease with a daily intake of 600 g of vegetables; Leenders et al [49] suggest an increase in longevity with fruit and vegetable consumption; and Chowdhury et al [50] report that individuals consuming a well-balanced diet are healthier with a strong immune system and have a reduced risk of contracting infectious diseases such as COVID-19.

On the basis of the aforementioned recommendations for healthy habits and the scientific evidence that supports each activity, 4 challenges are generated (summarized schematically in Textbox 2).

**Explanatory summary of the 4 proposed challenges.**

**Challenge 1**

- Name: High-intensity interval training (HIIT) *7-minute workout*
- Description: the challenge consists of performing 4 three-round sessions of the *HIIT 7-minute workout* in a week
- Included activities: *HIIT 7-minute workout*

**Challenge 2**

- Name: *Walk more than 10,000 steps every day*
- Description: the challenge consists of walking >10,000 steps every day of the week
- Included activities: walking >10,000 steps

**Challenge 3**

- Name: *Balance training*
- Description: the challenge consists of performing at least 2 days of eccentric training using gliding disks in a week
- Included activities: eccentric training protocol using gliding disks

**Challenge 4**

- Name: *Eat at least 5 servings of fruits and vegetables per day*
- Description: the challenge consists of eating at least 5 servings of fruits and vegetables every day of the week
- Included activities: eating at least 5 servings of fruits and vegetables

**Common information for all 4 challenges**

- Duration: 7 days
- Types of messages
  - Generic *gain framed*
  - Targeted *loss framed*
  - Tailored *gain framed*
  - Tailored and personalized *gain framed*

### Challenge 1: *High-Intensity Interval Training 7-Minute Workout*

This challenge consists of the user performing the *high-intensity interval training (HIIT) 7-minute workout* 4 days per week. The basis for this challenge comes from the WHO and PAG recommendation to combine aerobic PA and muscle-strengthening PA and from the training proposed in the study by Klika and Jordan [51], in which PA training is performed only with body weight aerobic PA and muscle strengthening [2,52]. The training consists of repeating 2 or 3 sets of the *HIIT 7-minute workout* [51]. On the basis of the WHO and PAG vigorous PA practice recommendations, in this challenge, the user will be asked to perform 3 sets daily of the *HIIT 7-minute workout* at least 4 times per week.

If no workout has been performed after 2 days from the start of the challenge, the user will receive “PA practice improves your physical and mental health” as a generic message to highlight the benefit of meeting their goals.

After 3 days from the start of the challenge without performing any training, the user will receive “Not performing your strength training sessions will worsen your health” as a targeted message framed to highlight the harms of not meeting the PA and strength training goals.

When the user has completed 2 training sessions, they will receive “Cheer up! You have been strength training this week, keep it up to improve your quality of life” as a tailored message framed around the benefit.

Finally, when the user reaches their goal of 4 strength workouts per week, they will receive “Great job, [name of user]! You’ve completed this challenge, keep it up—you’re decreasing your chance of getting heart disease by more than 10%!” as a personalized tailored message based on the virtues of performing PA.

### Challenge 2: *Walk More Than 10,000 Steps Every Day*

This challenge consists of the user walking >10,000 steps daily all 7 days of the week, based on the results of the recent systematic review and meta-analysis conducted by Jayedi et al [52], in which a clear decrease in the risk of all-cause mortality is observed when walking >10,000 steps daily, in addition to a 12% decrease in the risk of all-cause mortality with each increment of 1000 steps per day. The user will be asked to replace sedentary time with PA practice and walk >10,000 steps every day for 7 consecutive days.

After 1 day from the start of the challenge, if the user has not walked 10,000 steps, they will receive “The practice of PA reduces the risk of heart disease, hypertension, and type 2 diabetes” as a generic message to highlight the benefit of meeting their goals.

After 3 days from the start of the challenge without walking 10,000 steps, the user will receive “Not reaching your daily step goals will worsen your quality of life” as a targeted message framed to highlight the harms of not meeting daily step goals.

When the user has walked >10,000 steps for 4 consecutive days, they will receive “Cheer up! You have reached your daily goal again, keep it up to improve your cardiovascular health” as a tailored message framed around the benefit.

Finally, when the user manages to reach their daily step goal for 7 consecutive days, they will receive “Congratulations, [name of user]! You have completed this challenge, keep it up, you have just improved your physical and mental health!” as a personalized tailored message based on the virtues of performing PA.

### Challenge 3: *Balance Training*

This challenge consists of the user performing strength training ≥2 days per week. To meet this goal, the user will be asked to perform the eccentric training exercises using sliding disks [53] at least twice a week.

After 2 days from the start of the challenge without performing any training, the user will receive “PA practice increases your longevity” as a generic message to highlight the benefit of meeting their goals.

After 4 days from the start of the challenge without performing any training, the user will receive “By not performing your balance training you are increasing the probability of falling” as a targeted message framed to highlight the harms of not meeting their weekly training goal.

When the user has completed 1 workout, they will receive “Cheer up! You’ve had a workout this week, keep it up to improve your balance” as a tailored message framed around the benefit.

Finally, when the user reaches their goal of 4 strength workouts per week, they will receive “Great job, [name of user]! You’ve completed this challenge, keep it up, you’ve just improved your balance and bone health!” as a personalized tailored message based on the virtues of performing balance training.

### Challenge 4: *Eat at Least 5 Servings of Fruits and Vegetables per Day*

This challenge consists of the user eating at least 5 servings of fruits and vegetables per day, based on the results of the recent systematic review and meta-analysis conducted by Aune et al [6] as well as the recommendations of the WHO [11] and the FAO [7]. Both organizations recommend the consumption of at least 5 servings of fruits and vegetables per day, and Aune et al [6] report a 31% decrease in the risk of contracting diseases with a daily intake of 800 g of fruits and vegetables, a 19% decrease with a daily intake of 600 g of fruits, and a 25% decrease with a daily intake of 600 g of vegetables. To perform this challenge, the user will be asked to consume at least 5 servings of fruits and vegetables per day (1 serving is approximately 150 g) on all 7 days of the week.

After 1 day from the start of the challenge, if the user has not consumed at least 5 servings of fruits and vegetables, they will receive “WHO recommends the consumption of fruits and vegetables to reduce the risk of heart disease, hypertension, and type 2 diabetes” as a generic message to highlight the benefit of meeting their goals.

After 3 days from the start of the challenge without consuming the 5 daily portions, the user will receive “If you don’t eat at least five servings of fruits and vegetables a day, you increase your risk of disease” as a targeted message framed to highlight the harms of not meeting the daily goals.

When the user has reached the goal of eating at least 5 servings of fruits and vegetables a day for 4 consecutive days, they will receive “Cheers! You have reached your daily goal again, keep it up to increase your life expectancy” as a tailored gain-framed message.

Finally, when the user manages to reach their daily goal of eating at least 5 servings of fruits and vegetables for 7 consecutive days, they will receive “Congratulations, [name of user]! You have completed this challenge, keep it up, you are reducing the probability of being diagnosed with cancer by more than 10%!” as a personalized tailored message based on the virtues of consuming fruits and vegetables.

### Architectural Perspective

From an architectural perspective, the system is fundamentally constituted through a BC network. In this network, challenge-related achievements are stored, and their verification is performed using SCs. As mentioned in the previous sections, the objective of the system is to provide a motivating user experience so that participants feel engaged in the proposed activity and thus adhere to the challenges introduced in the system. By using this registration and verification tool, users can be assured of the veracity of their achievements.

To operate within the system, the user must make use of the dApp provided for this purpose. This application will run on the user’s local device and be responsible for managing the user’s identity and sending for publication on the BC network the data registered for the event in which the user is taking part. This monitoring of activities related to the challenge itself should be carried out in the least invasive way possible.

The BC network used for this purpose was hosted on an external service provider that runs the Hyperledger nodes with support for Web3 applications. In particular, tests were performed using support from Kaleido [54].

According to the proposed model, the SC defined for each challenge automates challenge-specific decision-making, performing tasks such as the following:

- Verifying the fulfillment of the conditions for each challenge: once the conditions for the challenge in question have been met, the established rewards are assigned.
- Generating the established messages: these messages correspond to certain challenge conditions that are analyzed by the SC. Thus, when a user does not perform the walking PA on a particular day, a corresponding alert is generated and sent to the user.

For the interaction with the BC network, the deployed nodes offer a representational state transfer application programming interface that allows the invocation of remote services in a simple way. A description of the most relevant procedures can be found in Table 2. The user self-authenticates when sending data by providing their public key and creating an encrypted field with their private key to validate the information sent. In other words, the access credentials will be managed only by the client device. It is also worth noting that any user or node can obtain a complete list of records in the chain and obtain the messages that have not yet been delivered.

**Table 2:** Description of the most relevant procedures.

Action	Resource	Purpose	Input parameters
POST	/v1/records	Add a challenge record	Challenge ID User log-in Public key Challenge facts Encrypted hash with private key
GET	/v1/records	Obtain the complete data string or the data referring to a log-in or challenge	User log-in (optional) Challenge (optional) Initial date (optional)
GET	/v1/awards	Obtain the rewards associated with a log-in	User log-in
GET	/v1/messages	Obtain a user’s pending messages	User log-in (optional) Initial date (optional)

As an example, in the case of challenge 1, *HIIT 7-minute workout*, the user must perform 3 sets of the 7-minute HIIT workout per day for at least 4 days per week. The user must manually record the sets performed each day using the dApp provided for this purpose. In addition, the user must attach a JPEG file demonstrating the completion of the training (eg, a screenshot of the heart rate variation intervals during the HIIT execution). Subsequently, the SC corresponding to this challenge, illustrated in Algorithm 1 within Textbox 3, performs the verification of the established conditions for this particular challenge. This mechanism supports the handling of messages sent to users as well as the allocation of a reward in the form of a transfer of the network’s own cryptocurrency as a reward.

The information, which is stored in the network, can be used as a support to encourage competition among participants. To this end, using these data, dashboards can be generated showing the most involved user in the activity—the one who has walked the most steps, performed the most sets of the *HIIT 7-minute workout*, or consumed the most number of servings of fruits and vegetables—or any other parameter that may be interesting and can be used to encourage participation. The idea is to achieve a critical mass of users among whom a habit of healthy activities is inculcated through this system of rewards and competition among peers.

Of note, the tests carried out in the laboratory after the implementation of the BC network showed satisfactory results in its functioning.

**Algorithm 1: smart contract snippet for checking the high-intensity interval training challenge.**

function checkNewAward(recordChallengeHIIT memory actualRecord) public returns (bool success) {     bool result = false;

     uint currentChain=0;

     uint userSearched = actualRecord.user;

     uint lastHiit = block.timestamp; // today     for (uint i = 0 ; i < recordCollection.length - 1; i++) {              if (recordCollection[i].user == userSearched) {                  if ( ((recordCollection[i].date) >= (lastHiit + (4 days ))) && ( (recordCollection[i].date) < (lastHiit + 3 days )) )

                  {

                  currentChain = currentChain + 8;

                  }

                  if ( ((recordCollection[i].date) >= (lastHiit + (3 days ))) && ( (recordCollection[i].date) < (lastHiit + 2 days )) )

                  {

                    currentChain = currentChain + 4;

                  }

                  if ((recordCollection[i].date >= (lastHiit + 2 days )) && (recordCollection[i].date < (lastHiit + 1 days )))

                  {

                    currentChain = currentChain + 2;

                  }

                  if ((recordCollection[i].date >= (lastHiit + 1 days )) && (recordCollection[i].date < lastHiit ))

                  {

                    currentChain = currentChain + 1;

                  }

              }

          if (currentChain==15)

          {

            success = true;

            awardChallengeHIIT memory data;

            data.date = block.timestamp;

            data.user = actualRecord.user;

            data.award = “Award”;

            addAward(data);

          }

     }

  return result;

   }

## Discussion

With regard to the objectives and hypotheses set out in this work, we have been able to create a tool that encourages healthy lifestyle habits in the population through challenges. BC technology will be key to the implementation of these habits and the monitoring of compliance in the least intrusive way and without the need to rely on trusted third parties.

### Limitations and Future Work

The limitations of this work include the limited consideration of General Data Protection Regulation (GDPR) implications and the manual need for information upload. In future work and to overcome the latter limitation, we propose the introduction of artificial intelligence techniques and the use of wearables connected to the dApp, a method similar to that used in the study by Santos-Gago et al [55].

### Comparison With Prior Work

Regarding the characteristics considered relevant in the 17 articles cited in the *Results* section, the following aspects are worth highlighting in comparison with our proposal.

Regarding the access policy, our platform is formed by a permissioned network. Therefore, only authorized nodes will be able to participate in the platform, as is the case with 3 (18%) of the 17 studies [33,34,36]. Other approaches [31,32,42,43,47] involved the use of a permissioned private BC, Rupasinghe et al [44] used a permissioned consortium BC, and Mulyati et al [40] used a public BC network. The other studies (7/17, 41%) do not indicate the type of network used.

Concerning SC, 8 (47%) of the 17 studies [35-40,45,46] did not report on their implementation, in contrast to our approach, which is similar to that of 9 (53%) of the 17 studies [31-34,41-44,47], which also made use of this special BC feature.

Regarding the use of cryptocurrencies to incentivize users, only Alsalamah et al [31] take advantage of this feature; the other works cited (16/17, 94%) do not indicate the use of cryptocurrencies. In our work, too, this feature is not exploited.

Concerning end-user delivery support, we implemented a dApp to be able to offer all the features that only BC can provide, similar to the approach used in 3 (18%) of the 17 studies [31,40,43].

Finally, regarding the use of oracles, NFTs, and the proposed PA and scientific evidence–based feeding, none of the aforementioned works indicate the use of these features. On the one hand, our proposal does not involve the use of oracles either. Nevertheless, according to the suggested model, it is possible to include oracles as actors with minor updates on the low level of the designed system. On the other hand, we use rewards to achieve a gamification experience and engage users in healthy lifestyle habits through challenges. We have based these challenges, composed of PA and healthy eating habits, on scientific evidence, supported by relevant organizations such as the WHO and the FAO.

### Conclusions

The emergence of disruptive technologies such as BC has opened the door to new possibilities in the provision of services to society. This work explores the potential of this technology in the development of services that improve people’s quality of life. To this end, strategies have been developed that allow, using gamification, the monitoring of adherence to new healthy habits in a simple way and, consequently, help to increase adherence.

The use of BC technology has been fundamental for meeting these objectives. In the review of previous works, it can be observed that the potential of BC has not been fully exploited. In our model, the aim is to show how to fully use this technology. Consequently, it is worth highlighting the following aspects of the platform:

- In an autonomous manner, without the need for supervision by a human agent and without the possibility of blockage, the verification of challenge completion is carried out. In the same process, the reward allocation is carried out, which cannot be interfered with by any system agent.
- All participants in the system can transparently verify the status of challenges at all times, thus increasing system transparency.
- As trust in the data resides in the network itself, there is no need to rely on a third party. This eliminates distrust because the promoter of the challenge is not known at first hand.

By contrast, when using this technology, there are certain deployment aspects that must be taken into account, including, primarily, the fact that future practitioners must be aware that once an SC is deployed, it cannot be modified, as could be the case with other technologies where the software is easily updatable. This is why it is very important to adequately test the system in development before deploying it in production.

This technology offers other elements that can improve users’ adherence to the system but have not yet been properly implemented in this prototype. We are talking about both the use of NFTs to reward the fulfillment of certain challenges or meta-challenges and the use of oracles for the unsupervised acquisition of information to eliminate the need for user input and improve SC decision-making.

Although the system is still pending functional validation in a realistic environment, the experimental result has been satisfactory. A simple tool has been generated for the user, with a scalable and inexpensive deployment for service providers and with great potential for improving people’s health. In this aspect, the adequate generation of training plans has played a fundamental role. These have been obtained from validated medical sources (eg, the WHO and the PAG) and therefore offer a high level of confidence.

A negative aspect of the system, pending more rigorous treatment, is compliance with the GDPR. This legal framework establishes a series of conditions, such as the elimination of user information when the user demands it. However, it should be noted that, in our proposal, no personal or medical data are recorded directly on the BC. The personal data are stored in the personal device, and the data regarding the completion of the different challenges are recorded on the BC. In fact, there are already critical voices regarding these aspects, and they are calling for a revision of the legal framework to facilitate the adoption of these new technologies [56].

In conclusion, a tool has been created through which healthy lifestyle habits can be inculcated in terms of both PA and healthy eating. Furthermore, it has been automated in the most transparent, safe, and least intrusive way possible using BC technology. Thereby, a tool to reduce the risk of all-cause mortality and to increase the well-being of society has been developed.

## Abbreviations

BC: blockchain
dApp: decentralized application
FAO: Food and Agriculture Organization
GDPR: General Data Protection Regulation
HIIT: high-intensity interval training
NFT: nonfungible token
OECD: Organization for Economic Co-operation and Development
PA: physical activity
PAG: physical activity guidelines for Americans
POA: proof of authority
POW: proof of work
SC: smart contract
WHO: World Health Organization

## References

[1] Bull FC, Al-Ansari SS, Biddle S, Borodulin K, Buman MP, Cardon G, Carty C, Chaput J, Chastin S, Chou R, Dempsey PC, DiPietro L, Ekelund U, Firth J, Friedenreich CM, Garcia L, Gichu M, Jago R, Katzmarzyk PT, Lambert E, Leitzmann M, Milton K, Ortega FB, Ranasinghe C, Stamatakis E, Tiedemann A, Troiano RP, van der Ploeg HP, Wari V, Willumsen JF (2020). World Health Organization 2020 guidelines on physical activity and sedentary behaviour. Br J Sports Med.

[2] Piercy KL, Troiano RP, Ballard RM, Carlson SA, Fulton JE, Galuska DA, George SM, Olson RD (2018). The physical activity guidelines for Americans. JAMA.

[3] World Health Organization (2020). WHO Guidelines on Physical Activity and Sedentary Behaviour: Web Annex: Evidence Profiles.

[4] Yang Y, Dixon-Suen S, Dugué P-A, Hodge A, Lynch B, English D (2022). Physical activity and sedentary behaviour over adulthood in relation to all-cause and cause-specific mortality: a systematic review of analytic strategies and study findings. Int J Epidemiol.

[5] Barrera J (2003). Actividad física como estilo de vida saludable: criterios básicos. Revista Médica de Risaralda.

[6] Aune D, Giovannucci E, Boffetta P, Fadnes LT, Keum N, Norat T, Greenwood DC, Riboli E, Vatten LJ, Tonstad S (2017). Fruit and vegetable intake and the risk of cardiovascular disease, total cancer and all-cause mortality-a systematic review and dose-response meta-analysis of prospective studies. Int J Epidemiol.

[7] (2020). Frutas y verduras – esenciales en tu dieta Año Internacional de las Frutas y Verduras, 2021. Documento de antecedentes.

[8] Hooper L, Abdelhamid A, Bunn D, Brown T, Summerbell C, Skeaff C (2015). Effects of total fat intake on body weight. Cochrane Database Syst Rev.

[9] Nishida C, Uauy R (2009). WHO Scientific Update on health consequences of trans fatty acids: introduction. Eur J Clin Nutr.

[10] World Health Organization (2003). Diet Nutrition and the Prevention of Chronic Diseases Report of a Joint WHO/FAO Expert Consultation.

[11] World Health Organization (2003). Food-based dietary guidelines in the WHO European Region. WHO Regional Office for Europe.

[12] World Health Organization (2015). Guideline: Sugars Intake for Adults and Children.

[13] Williamson C, Kelly P, Tomasone JR, Bauman A, Mutrie N, Niven A, Richards J, Baker G (2021). A modified Delphi study to enhance and gain international consensus on the Physical Activity Messaging Framework (PAMF) and Checklist (PAMC). Int J Behav Nutr Phys Act.

[14] Williamson C, Baker G, Mutrie N, Niven A, Kelly P (2020). Get the message? A scoping review of physical activity messaging. Int J Behav Nutr Phys Act.

[15] Conroy DE, Hojjatinia S, Lagoa CM, Yang C, Lanza ST, Smyth JM (2019). Personalized models of physical activity responses to text message micro-interventions: a proof-of-concept application of control systems engineering methods. Psychol Sport Exerc.

[16] Latimer AE, Brawley LR, Bassett RL (2010). A systematic review of three approaches for constructing physical activity messages: what messages work and what improvements are needed?. Int J Behav Nutr Phys Act.

[17] Bassett RL, Ginis Kathleen A Martin (2011). Risky business: the effects of an individualized health information intervention on health risk perceptions and leisure time physical activity among people with spinal cord injury. Disabil Health J.

[18] Bassett-Gunter RL, Martin Ginis KA, Latimer-Cheung AE (2013). Do you want the good news or the bad news? Gain- versus loss-framed messages following health risk information: the effects on leisure time physical activity beliefs and cognitions. Health Psychol.

[19] Lin I, Liao T (2017). A survey of blockchain security issues and challenges. Int J Netw Secur.

[20] Kim J-W (2021). Analysis of blockchain ecosystem and suggestions for improvement. J Inform Commun Convergence Eng.

[21] Monrat AA, Schelen O, Andersson K (2019). A survey of blockchain from the perspectives of applications, challenges, and opportunities. IEEE Access.

[22] Oyinloye DP, Teh JS, Jamil N, Alawida M (2021). Blockchain consensus: an overview of alternative protocols. Symmetry.

[23] Hussien HM, Yasin SM, Udzir NI, Ninggal MI, Salman S (2021). Blockchain technology in the healthcare industry: trends and opportunities. J Industrial Inform Integration.

[24] Bukhari D (2020). Blockchain technology: a bibliometric analysis. HCI International 2020 - Posters.

[25] Lopez-Barreiro J, Alvarez-Sabucedo L, Garcia-Soidan JL, Santos-Gago JM (2022). Use of blockchain technology in the domain of physical exercise, physical activity, sport, and active ageing: a systematic review. Int J Environ Res Public Health.

[26] Buterin V (2013). Ethereum White Paper.

[27] Beniiche A (2020). A study of blockchain oracles. arXiv.

[28] Cai W, Wang Z, Ernst JB, Hong Z, Feng C, Leung VC (2018). Decentralized applications: the blockchain-empowered software system. IEEE Access.

[29] Gómez-Díaz R, García-Rodríguez A (2018). Bibliotecas, juegos y gamificación: una tendencia de presente con mucho futuro. ThinKEPI.

[30] Miau S, Yang J (2018). Bibliometrics-based evaluation of the Blockchain research trend: 2008 – March 2017. Technol Analysis Strategic Manage.

[31] Alsalamah HA, Nasser S, Alsalamah S, Almohana AI, Alanazi A, Alrrshaid F (2021). Wholesome coin: a pHealth solution to reduce high obesity rates in gulf cooperation council countries using cryptocurrency. Front Blockchain.

[32] Frikha T, Chaari A, Chaabane F, Cheikhrouhou O, Zaguia A (2021). Healthcare and fitness data management using the IoT-based blockchain platform. J Healthc Eng.

[33] Jamil F, Kahng HK, Kim S, Kim D (2021). Towards secure fitness framework based on IoT-enabled blockchain network integrated with machine learning algorithms. Sensors (Basel).

[34] Jamil F, Qayyum F, Alhelaly S, Javed F, Muthanna A (2021). Intelligent microservice based on blockchain for healthcare applications. Comput Material Continua.

[35] Cao P, Zhu G, Zhang Q, Wang F, Liu Y, Mo R (2021). Blockchain-enabled HMM model for sports performance prediction. IEEE Access.

[36] Hong Y, Park D-w (2020). Big data and blockchain to improve performance of professional sports teams. ASM Sci J.

[37] Yu S (2021). Application of blockchain-based sports health data collection system in the development of sports industry. Mobile Inform Syst.

[38] Ma F (2021). Design of running training assistance system based on blockchain technology in wireless network. J Wireless Com Network.

[39] Shan Y, Mai Y (2020). Research on sports fitness management based on blockchain and Internet of Things. J Wireless Com Network.

[40] Rahardja U, Hardini M, Al Nasir AL, Aini Q, Mulyati (2020). Taekwondo sports test and training data management using blockchain. Proceedings of the 2020 Fifth International Conference on Informatics and Computing (ICIC).

[41] Khezr S, Benlamri R, Yassine A (2020). Blockchain-based model for sharing activities of daily living in healthcare applications. Proceedings of the 2020 IEEE Intl Conf on Dependable, Autonomic and Secure Computing, Intl Conf on Pervasive Intelligence and Computing, Intl Conf on Cloud and Big Data Computing, Intl Conf on Cyber Science and Technology Congress (DASC/PiCom/CBDCom/CyberSciTech).

[42] Rahman MA, Hossain MS, Loukas G, Hassanain E, Rahman SS, Alhamid MF, Guizani M (2018). Blockchain-based mobile edge computing framework for secure therapy applications. IEEE Access.

[43] Rahman M, Abualsaud K, Barnes S, Rashid M, Abdullah S (2020). A natural user interface and blockchain-based in-home smart health monitoring system. Proceedings of the 2020 IEEE International Conference on Informatics, IoT, and Enabling Technologies (ICIoT).

[44] Rupasinghe T, Burstein F, Rudolph C, Strange S (2019). Towards a blockchain based fall prediction model for aged care. Proceedings of the Australasian Computer Science Week Multiconference.

[45] Silva CA, Aquino GS, Melo SR, Egídio DJ (2019). A fog computing-based architecture for medical records management. Wireless Commun Mobile Computing.

[46] Spinsante S, Poli A, Mongay Batalla J, Krawiec P, Dobre C, Bǎjenaru L, Mavromoustakis CX, Costantinou CS, Molan G, Herghelegiu AM, Prada GI, Drǎghici R, González–Vélez H (2021). Clinically-validated technologies for assisted living. J Ambient Intell Human Comput.

[47] Velmovitsky PE, Miranda PA, Vaillancourt H, Donovska T, Teague J, Morita PP (2020). A blockchain-based consent platform for active assisted living: modeling study and conceptual framework. J Med Internet Res.

[48] Momma H, Kawakami R, Honda T, Sawada SS (2022). Muscle-strengthening activities are associated with lower risk and mortality in major non-communicable diseases: a systematic review and meta-analysis of cohort studies. Br J Sports Med.

[49] Leenders M, Sluijs I, Ros MM, Boshuizen HC, Siersema PD, Ferrari P, Weikert C, Tjønneland A, Olsen A, Boutron-Ruault M, Clavel-Chapelon F, Nailler L, Teucher B, Li K, Boeing H, Bergmann MM, Trichopoulou A, Lagiou P, Trichopoulos D, Palli D, Pala V, Panico S, Tumino R, Sacerdote C, Peeters PH, van Gils CH, Lund E, Engeset D, Redondo ML, Agudo A, Sánchez MJ, Navarro C, Ardanaz E, Sonestedt E, Ericson U, Nilsson LM, Khaw K, Wareham NJ, Key TJ, Crowe FL, Romieu I, Gunter MJ, Gallo V, Overvad K, Riboli E, Bueno-de-Mesquita HB (2013). Fruit and vegetable consumption and mortality: European prospective investigation into cancer and nutrition. Am J Epidemiol.

[50] Chowdhury MA, Hossain N, Kashem MA, Shahid MA, Alam A (2020). Immune response in COVID-19: a review. J Infect Public Health.

[51] Klika B, Jordan C (2013). High-intensity circuit training using body weight: maximum results with minimal investment. ACSM's Health Fitness J.

[52] Jayedi A, Gohari A, Shab-Bidar S (2022). Daily step count and all-cause mortality: a dose-response meta-analysis of prospective cohort studies. Sports Med.

[53] Lopez-Barreiro J, Hernandez-Lucas P, Garcia-Soidan JL, Romo-Perez V (2021). Effects of an eccentric training protocol using gliding discs on balance and lower body strength in healthy adults. J Clin Med.

[54] Kaleido.

[55] Santos-Gago JM, Ramos-Merino M, Alvarez-Sabucedo LM (2021). Identification of free and WHO-compliant handwashing moments using low cost wrist-worn wearables. IEEE Access.

[56] Noh JH, Kwon HY (2019). A study on smart city security policy based on blockchain in 5G Age. Proceedings of the 2019 International Conference on Platform Technology and Service (PlatCon).

